# The Useful Life of Reinforced Concrete Structures with Reinforcement Corrosion Due to Carbonation in Non-Aggressive and Normal Exposures in the Spanish Mediterranean

**DOI:** 10.3390/ma15030745

**Published:** 2022-01-19

**Authors:** Pascual Saura-Gómez, Carlos Rizo-Maestre, Víctor Echarri-Iribarren

**Affiliations:** Departamento de Construcciones Arquitectónicas, Universidad de Alicante, San Vicente, 03690 Alicante, Spain; carlosrm@ua.es (C.R.-M.); victor.echarri@ua.es (V.E.-I.)

**Keywords:** corrosion, carbonation, start life, propagation life, useful life, environment

## Abstract

Some reinforced concrete structures must be repaired at an early stage in their life due to the oxidation processes suffered by their reinforcements; such processes involve serious pathologies that affect the stability and safety of buildings. Spanish legislation distinguishes several classes of environments, with non-aggressive and normal exposure providing a longer useful life of the structure. The present study shows that some structural elements in reinforced concrete, mainly the pillars in the area of contact with the ground, are exposed to significant corrosion by carbonation. This position of the structural elements dramatically and abruptly shortens the useful life of the models provided for the current regulations. A total of 17 pillars in 10 buildings of different ages and locations in the Spanish Mediterranean area, not subject to the presence of chlorides, have been analyzed. These buildings are situated in environments considered by the standard as normal and non-aggressive. The actual carbonation that these elements present have been compared with that which can be derived from the model established by Spanish regulations. Of these pillars, 14 present a carbonation higher than that derived from the model, and the last three pillars largely conform to the figures of the model. This significant deviation shows the need for a revision of the Spanish EHE 08 regulation, which should include aspects such as the action of dampness by capillarity and the differences in electrochemical potential between the different materials.

## 1. Introduction

### 1.1. Background

One of the most frequent pathologies in reinforced concrete is the corrosion of the reinforcements. A large part of the residential buildings in Spain and in many other developed countries includes reinforced concrete structures that are regulated by specific regulations, which, in most cases, require a building life greater than or equal to 50 years. The extensive use of concrete throughout the 20th century has increased the levels of definition (strength, consistency, maximum aggregate size and exposure environment) and quality control in its manufacturing and implementation. For two millennia, the concrete prepared by the Romans using lime, pozzolana and aggregates has survived the natural elements and external actions, proving its durability. With the use of concrete, one has the idea of a very durable material, given its stone-like characteristics and mechanical qualities. Concrete guarantees a very suitable medium for the protection of reinforcements thanks to its high alkalinity (pH = 12–13), acting as a chemical barrier, and the physical barrier that it provides.

**Figure 1:**
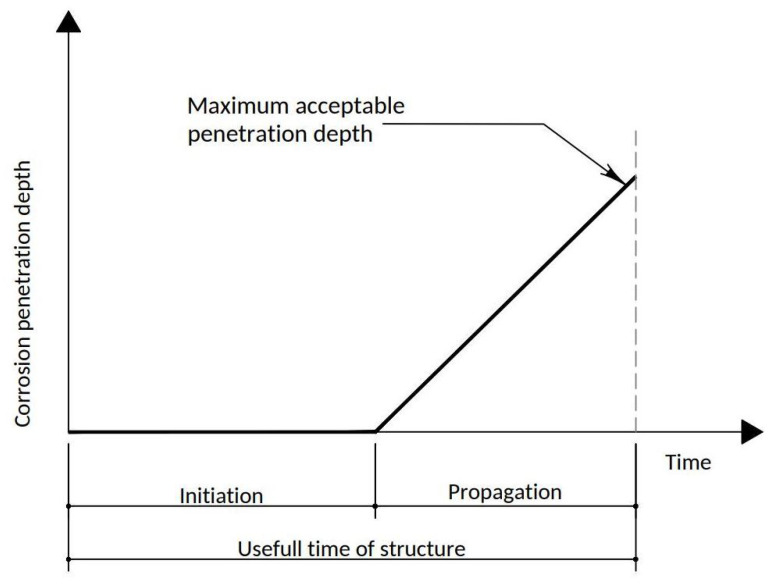
Initiation and propagation periods for corrosion in a reinforced concrete structure (Tuutti model) [14].

However, under aggressive conditions (generally related to carbonation or the presence of chlorides) [1,2,3,4,5,6,7,8], even concrete that has been prepared and applied correctly can lose its protective properties and allow reinforcements to corrode before the minimum of 50 years of its expected useful life have elapsed, sometimes with serious consequences. Therefore, this concept of almost eternal durability is no longer applicable, and indeed the need for continuous maintenance of concrete structures is now recognized, including action and repair on structures with an age of less than 10 years. Reinforced concrete structures have become widespread, especially from the second half of the 20th century onwards, and they are present in a large percentage of our residential buildings. Currently, there is an awareness of the importance of this pathology since there are structures that are old enough in order to confirm the problems caused by corrosion. The deterioration of the structures motivates a constant concern for economic losses due to the shortening of the useful life of buildings. Starting in 1980, research activity on this problem increased with the analysis of the physiological aspects of the behavior of steel in concrete, such as the solutions present in the micropores of the concrete that surround the steel, the electrochemistry of steel and the protection mechanisms for steel in concrete (passivating layer). Progress has been made in understanding the phenomena and mechanisms of corrosion, but this pathology continues to seriously affect buildings, and therefore, there is a real interest in extending our knowledge of the corrosion process [4,9,10,11,12,13], the first phase of which is the initiation of corrosion, in which the reinforcement is passivated, but some phenomena can lead to the loss of passivity, such as carbonation or the penetration of chlorides in the concrete covering. The second phase is the propagation of corrosion, which begins when the steel depassivates and ends when a limit state is reached in which the consequences of corrosion can lead to the end of the service life of the structure (Figure 1) [14,15,16,17].

The standards and instructions for the preparation of reinforced concrete consider several general classes of exposure related to the corrosion of reinforcements: non-aggressive, normal, marine and with chlorides other than the marine environment. In non-aggressive environments (interiors of buildings) and normal environments (with medium and high humidity but with initial corrosion not related to chlorides), the prediction models for the useful life of the structure give very high figures, in the order of more than 100 years.

However, there are numerous cases of premature reinforcement corrosion in reinforced concrete elements in these types of environments with a low level of aggressiveness. Therefore, there is a need to analyze the conditions existing in each one of them in order to determine the common factors that may contribute to the corrosion and which are not currently considered by the regulations since the predictions made using the corresponding models are so inaccurate. In these environments with no or low levels of chlorides, the initiation and propagation of steel corrosion in reinforced concrete can only occur due to carbonation in the rebar coating.

**Figure 2:**
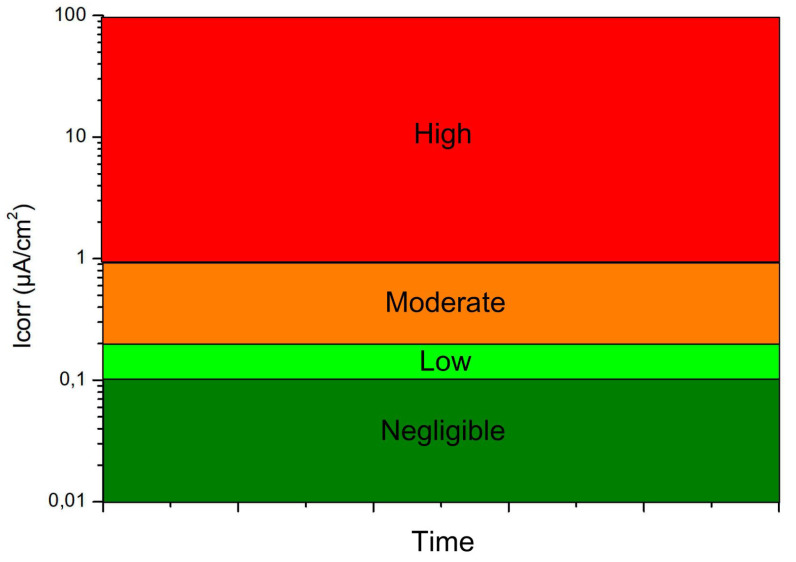
Corrosion rate classification diagram.

The corrosion rate of steel is normally expressed as the rate of penetration of generalized corrosion (thickness of the steel reinforcement that is converted to oxide per unit of time) and is measured in μm/year. Often, especially in laboratory tests, it is expressed in electrochemical units as mA/m^2^ or μA/cm^2^. In the case of steel, 1 mA/m^2^ or 0.1 μA/cm^2^ corresponds to a mass loss of approximately 90 g/m^2^/year and a penetration rate of approximately 11.7 μm/year. The corrosion rate can be considered negligible if it is below 0.1 μA/cm^2^, low if it is between 0.1 and 0.2 μA/cm^2^, moderate between 0.2 and 1 μA/cm^2^ and high if it is between 1 and 100 μA/cm^2^ according to the RILEM (International Union of Laboratories and Experts in Construction Materials, Systems and Structures) (Figure 2).

### 1.2. State of Affairs

The first regulation on reinforced concrete in Spain was the IH-39 Instruction for Projects and the Execution of Concrete Works of the year 1939 [18], which regulated the use of materials, quantities and resistances, and in which the obligation of a minimum covering of 1 cm or a diameter of the reinforcement was already prescribed, and at least 3 cm for elements exposed to rain. Subsequently, IH-44 [19] came into force with the review carried out by a commission of which Eduardo Torroja was a member. After this, the Eduardo Torroja Institute of Construction and Cement drafted the Special Instruction for reinforced concrete structures HA-58 and HA-61. Subsequent instructions were EH-68 [20], EH-73 [21], EH-80 [22] and EH-82 [23], which increased the minimum coverage to 15 mm in coated walls or protected environments. After EH-88 [24], the standard incorporated different types of environments: I, interiors of buildings or exterior environments with low humidity, defining a minimum covering of 20 mm; II, normal, non-aggressive exterior structures with coatings greater than 30 mm; and III, aggressive, industrial or marine environments with coatings greater than 40 mm. In all cases, a minimum is also determined for the characteristic resistance of the concrete and the water/cement ratio. The regulations included in EH-91 [25] were very similar to the previous ones. It was not until EHE-98 for structural concrete elements [26] came into force that exposure classes similar to the current ones of EHE-08 [27] were defined, together with a minimum characteristic resistance of 25 N/mm^2^ (HA-25) for concrete in reinforced concrete elements. The general classes of exposure are: non-aggressive (I), normal (IIa: high humidity, and IIb: medium humidity), Marine (IIIa: aerial, IIIb: submerged and IIIc: in the tidal race zone and in the splash zone), and with chlorides not originating from the marine environment (IV). In addition, there are other specific classes of exposure: Aggressive chemical (Qa: weak, Qb: medium and Qc: strong), with frost (H with flux salts and F without flux salts) and Erosion (E). However, the general exposure classes are I (Non-aggressive) and II (normal), which, in the absence of aggressive agents, should provide a longer useful life according to the current EHE models [27,28,29]. In Eurocode 2, exposure classes are related to environmental conditions in accordance with EN 206-1; 1. No risk of corrosion or attack; 2. Corrosion induced by carbonation; 3. Corrosion induced by chlorides; 4. Corrosion induced by chlorides from seawater; 5. Freeze/Thaw Attack; 6. Chemical attack. According to this normative, the cases studied correspond to environment 2, corrosion induced by carbonation.

## 2. Objectives

Under normal service conditions, reinforcement corrosion does not usually occur in reinforced concrete, thanks to the high pH in the concrete’s pores. In this medium, the steel is surrounded by a layer of oxidation products, called a passivating layer, which in theory preserves it against corrosion indefinitely. However, this state of passivation of the reinforcements can be modified by certain chemical processes.

The main objective of this research work was the extent of the action that produces corrosion in structural elements of reinforced concrete where this should not occur [30,31]. The two areas on which the work has focused are the following:

- The detection, measurement and analysis of corrosion processes in reinforced concrete elements in non-aggressive and normal environments where corrosion by chlorides does not occur. For this purpose, a sample of ten buildings was analyzed, studying elements of their structure damaged by the corrosion of their rebars.
- The application of the EHE instruction regulating the execution and control of structural concrete elements for the studied environments that considers a certain start time for the corrosion process, simulating the conditions that occur in the coating of the reinforcement in an attack by carbonation up to the initial state of the propagation process on the rebar, and later, when it is in its propagation period, that is, in full development. In this phase, and for the media studied, it is necessary to consider the decrease in pH at the level of the steel-concrete interface produced by the hydrolysis of Fe^++^ions, resulting in the oxidation of the steel, which contributes to the increase in the speed of the cathodic process.

This research set out to analyze several elements of the reinforced concrete in the 10 buildings studied, which were in non-aggressive and normal environments, but in which corrosion had occurred, making it necessary to perform a rehabilitation and/or reinforcement intervention on the element since it had reached the end of its useful life. Carbonation started at the concrete surface and had gradually moved toward interior areas; the alkalinity of the concrete was neutralized by the carbon dioxide in the atmosphere, so the pH of the solutions present in the micropores of the concrete decreased to values below 9, and the passivating layer lost the alkaline protection condition that it should provide; thus, allowing the corrosion process. Once the steel has lost its protection, the spread of corrosion can occur if the necessary water and oxygen are present on the surface of the rebars.

The main consequences of the corrosion attack observed appear on the external surface of the concrete due to the damage to the concrete covering produced by the expansion of the corrosion products. These products, in fact, occupy a much larger volume than the original steel. The volume of corrosion products can be from two to six times greater than that of the iron from which they come, depending on their composition and the degree of hydration.

For example, the volumes of Fe_3_O_4_, Fe(OH)_2_, Fe(OH)_3_ and Fe(OH)_3_·3H_2_O [32] are, respectively, two, three, four and six times higher than that of iron. In general, the volume of corrosion products, a mixture of these oxides, hydroxides and oxyhydroxides, can be considered 3–4 times greater than that of iron [33].

In all the structural elements analyzed in the 10 buildings considered in this study, the corrosion of the reinforcements had occurred with greater or lesser losses in the reinforcement section (high corrosion rates), and all of them were in contact with the foundation (plumbing of pillars), although their relative situations with respect to the rest of the structure, the soil conditions, manufacturing, resistance, etc., were different.

In order to address the main objective of this research, these possible phases in the corrosion of reinforcement have been modeled, with the study of other objectives in each of the cases:

- Study of the initiation functions of carbonation corrosion.
- Study of the propagation functions of corrosion by carbonation.
- Theoretical depth of carbonation according to the age of the structure.
- Analysis of the factors that influence the process.
- Evolution of the corrosion rate [34].

The aim of this study was to discover the behavior of the elements studied and ultimately the possible generalization of these pathologies due to the corrosion of reinforcements in certain conditions and situations related to the structure that is beyond the provisions of the current EHE 08 standard [27].

## 3. Methodology

### 3.1. Structural Elements in the Different Exposure Environments

All the buildings included in this research had suffered corrosion processes in the reinforcements of some of their structural elements that had caused pathologies that ended their useful life and required a repair intervention [35]. The ten buildings are located in the provinces of Alicante and Murcia (south-east Spain), at the following addresses: 1D Calle General Pastor, 19, Dolores; 2G Calle San Pedro, 38, Guardamar del Segura; 3T Calle Ulpiano,71, Torrevieja; 4T Avenida de los Holandeses, Solmar building, Torrevieja; 5T Avenida de Inglaterra, 31, Maravillas building, Torrevieja; 6D Avenida de Crevillente, 4, Dolores; 7R Plaza de Héroes de Africa, Rojales; 8A Avenida de La Peseta, 19, Alicante; 9M Calle Iberia, 33, Building Torre Brisa, Aguilas; 10E Avenida de San Bartolomé de Tirajana, 8, Elche.

Prior to studying the different projects and as a necessary methodology for decision-making in the drafting of the execution projects, in all cases, the compressive strength and carbonation depth tests of the concrete element analyzed were carried out (two pillars in seven buildings and one pillar in three buildings, making a total of seventeen pillars) by approved quality control laboratories. All the studies were performed on the ground floor or basement pillars and in non-aggressive and normal environments, which are defined in Table 8.2.2. of EHE-08 [27]: General exposure classes related to rebar corrosion.

### 3.2. Data Collection from Experiments

**Figure 3:**
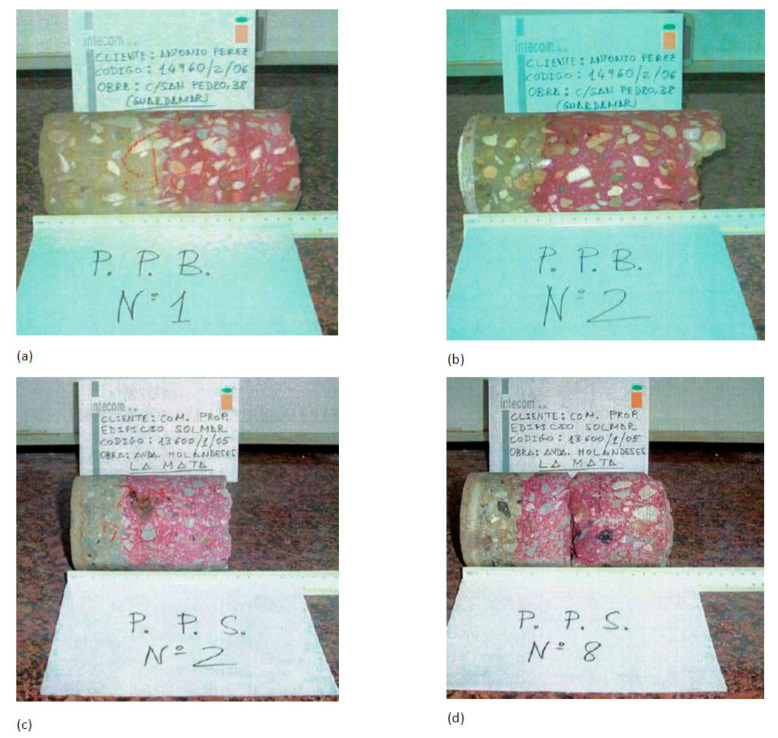
Depth of carbonation. (**a**) Building 2 Pillar 1. (**b**) Building 2 Pillar 2 (**c**) Building 4 Pillar 2. (**d**) Building 4 Pillar 8.

The data provided by the laboratories were:

- Compression resistance tests. In all the seventeen elements studied, and in each case for the corresponding laboratory, we proceeded with the extraction of test specimen controls by means of a 75 mm diameter rotating probe, in accordance with the UNE-EN 12504-1 and UNE-EN 12390-3 standards to determine the compressive strength of the concrete of the said elements.
- In many cases, non-destructive information tests were carried out by measuring the speed of ultrasound propagation in the elements with specimen extraction and in others to know their resistance from a comparative study. This information has not been used in this article and is the subject of future research.
- Carbonation depth test, taking a sample from the reinforced concrete element and applying an alcoholic phenolphthalein solution that produces a pink hue if the sample is not carbonated (pH greater than 9). Simple visual inspection reveals the carbonate area as the one that remains colorless (Figure 3).

Determination of the chloride content. In all the cases analyzed, the presence of chlorides was found to be negligible, which confirms that these were normal environments or protected from marine exposure.

The data gathered on-site by the project management of the works and the representative of the contracting companies were:

- **Coating of concrete.** Given the conditions of execution of the element (verticality, irregularities, absence of spacers, state of the reinforcements) and the measurement errors by the flexometer of +/−5 mm, minimum coverings of 20 mm were considered in all cases.
- **Diameter of the reinforcements**. In all the columns, the behavior of both the transverse reinforcements (6 and 8 mm in diameter) and the longitudinal reinforcements (16 and 20 mm in diameter) was studied. A total of 34 trusses in 17 columns were analyzed.
- **Building age**. All the buildings are the product of extensive professional experience in the Spanish Mediterranean area, with the examples studied having ages from 9 to 44 years, and they can be considered a representative sample of buildings of their type. Their age was obtained from the data provided by the property and checked with the construction date that appears in the land registry file (Virtual Headquarters of the Catastro) in each case. They mostly date back to the 1970s, when the regulations for concrete stipulated characteristic strengths of 150 Kg/cm^2^ and minimum coverings greater than 10 mm or the diameter of the longitudinal bars.

To carry out the present study of elements of reinforced concrete with corrosion of the reinforcements, whose aggressive environment is produced by the carbonation of the concrete, two periods must be distinguished: initiation and propagation of corrosion.

According to Annex 9 of EHE [27], point 1.2, the durability model for corrosion processes considers that the total time necessary for the attack or degradation to be significant is expressed as T = t${}_{i}$ + t${}_{p}$, where t${}_{i}$ is the corrosion initiation period and t${}_{p}$ is the propagation period.

### 3.3. Carbonation Corrosion

In damp environments, the carbon dioxide present in the air forms an acidic aqueous solution that can react with the hydrated cement paste and neutralize the alkalinity of the concrete (this process is known as carbonation).

The alkaline constituents of concrete are present in pore solutions (mainly as sodium and potassium hydroxides) but also in solid hydration products, such as Ca (OH)${}_{2}$ or CSH gel. Calcium hydroxide is the hydrate in cement paste that reacts most quickly with CO${}_{2}$. The reaction, which takes place in aqueous solutions, can be described as:

$${\mathrm{CO}}_{2}+{\mathrm{Ca}(\mathrm{OH})}_{2\;}=>{\mathrm{CaCO}}_{3}+H_{2}O$$

Carbonation does not cause any damage to concrete on its own. In fact, in the case of concrete made with Portland cement, it can even reduce porosity and lead to an increase in strength. However, carbonation has important effects on the corrosion of steel in concrete. The first consequence is that the pH of the pore solution falls from its normal value of pH 13–14 to values that approach neutrality. The second consequence of carbonation is that chlorides fixed in the form of hydrated calcium chloroaluminate and fixed to other hydrated phases can be released, making the solution in the pores even more aggressive [14,36,37,38,39,40]. In the cases studied, the level of chlorides was negligible, but this behavior is being analyzed for future research.

The carbonation reaction begins on the external surface and penetrates into the concrete, producing a low pH front. By testing the alcoholic phenolphthalein solution on a freshly fractured face, the areas where the pH is higher than 9 take on a pink color that is typical of phenolphthalein in basic environments, while the color of the carbonate areas remains unchanged (grayish concrete).

The rate of carbonation decreases with time, as the CO${}_{2}$ has to diffuse through the pores of the already carbonated outer layer. The penetration of carbonation as a function of time can be described as: x = K${}_{co2}t^{1/n}$ [14,27,41,42], where x is the depth of carbonation (mm), and t is the time (years). The exponent n is often approximately two, and therefore, this can be considered a parabolic trend: x = K${}_{CO2}$·t${}^{1/2}$. The coefficient of carbonation K${}_{CO2}$ (mm/year${}^{1/2}$) can be taken as a measure of the rate of penetration of carbonation for concrete and given environmental conditions. In dense and/or wet concrete, however, the reduction in carbonation rate over time is greater than that described by the parabolic formula, such that n > 2; in highly waterproofed concrete, the carbonation rate tends to be negligible after a certain time. In this research, the model regulated by the Annex 9 of the EHE standard has been considered (n = 2) [27].

The carbonation rate depends on both environmental factors (humidity, temperature, carbon dioxide concentration) and factors related to concrete (mainly its alkalinity and permeability) [43,44].

**Figure 4:**
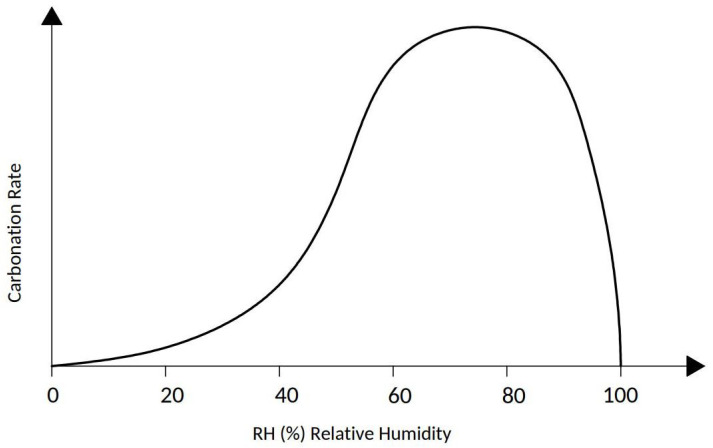
Schematic representation of the carbonation rate of concrete as a function of the relative humidity of the medium, under equilibrium conditions [14].

(a) Humidity. The rate of carbonation varies with the humidity of the concrete for two reasons. First, the diffusion of carbon dioxide in concrete is facilitated in the pores with air, but it is very slow through the pores filled with water (the diffusion of CO${}_{2}$ in water is four orders of magnitude slower than in air). The diffusion rate of CO${}_{2}$ decreases consequently with increasing concrete moisture until it is minimized when the concrete is saturated with water. This means that when concrete is wet, CO${}_{2}$ penetrates very slowly into it. On the other hand, the carbonation reaction takes place only in the presence of water so that it becomes negligible when the concrete is dry. The carbonation rate, and therefore, the K${}_{{CO}_{2}}$ value, will change from a humid climate to a dry climate. Under equilibrium conditions with an environment of constant relative humidity, the carbonation rate can be correlated with the humidity of the medium, as shown in Figure 4 [41,45,46]. The most critical relative humidity range for promoting carbonation is 60% to 80%. The EHE standard considers this condition in terms of the environment since it differentiates interior elements that are ventilated or not (relative humidity greater than 65% or condensation), and exterior elements with average annual precipitation greater than and less than 600 mm (Table 1). The models take into account an ambient coefficient (1 if the element is protected from rain and 0.5 if it is exposed).

(b) Concentration of CO_2_. The concentration of carbon dioxide in the atmosphere can vary from 0.03% in rural areas to more than 0.1% in urban areas. Comparatively high concentrations can be achieved under specific exposure conditions, such as inside garages or areas with motor vehicles. As the CO_2_ content of the air increases, the rate of carbonation increases [41]. Some researchers suggest that with a high concentration of CO_2_, the porosity of artificially carbonated concrete is greater than that obtained by natural exposure. However, this is controversial, as other studies have shown that even with 100% CO_2_ overpressure, the same microstructure occurs as in natural carbonation [47]. All the buildings studied are located in natural and/or urban environments in areas with little traffic and adequately ventilated; only three of the buildings had basements for parking or vehicle traffic, but in all cases, they were well ventilated and with minimal occupancy.

(c) Temperature. All other conditions being equal, especially humidity, which is, in general, the most important parameter, an increase in temperature will increase the carbonation rate. All the cases are in a uniform climatic zone with mild temperatures throughout the year (between 0 °C and 40 °C).

(d) Concrete composition and curing. The permeability of concrete is of great importance in the diffusion of carbon dioxide and, therefore, in the rate of carbonation. A decrease in the water/cement ratio (w/c) slows down the penetration of carbonation [48] since it reduces the capillary porosity of the hydrated cement paste. However, the advantages of a lower (w/c) ratio can only be achieved if the concrete is properly cured, as poor curing hinders the hydration of the cement paste and leads to a more porous cement matrix. Insufficient curing will mainly affect the porosity of the covering concrete, that is, the part that is intended to protect the reinforcement. In fact, the outermost layer of the concrete is the part most susceptible to water evaporation (worsening curing). This variable is impossible to control in an element that has already been produced, which is the case here. Therefore, the model considers directly related properties, such as the air coefficient and the average resistance of concrete to compression, which is always the result of a certain w/c ratio.

(e) Type of cement. The type of cement also influences the carbonation rate. In fact, the ability of concrete to fix CO${}_{2}$ is proportional to the alkalinity of its cement paste. For mixed cement, the hydration of pozzolanic materials or slag leads to a lower Ca(OH)${}_{2}$ content in the hardened cement paste, which can increase the carbonation rate. The denser structure produced by the hydration of mixed cement can slow down the diffusion of CO${}_{2}$. The lower alkalinity of cements with additions of fly ash or blast furnace slag can be compensated for by the lower permeability of their cement pastes if properly cured [48]. The condition of the type of cement has also been considered in the model with parameters depending on the type of binder.

### 3.4. Initiation of Corrosion Period

t${}_{i}$ = Corrosion initiation period, understood as the time it takes for the aggressive front to reach the rebar and cause the onset of corrosion.

t${}_{p}$ = Propagation of corrosion period, up to significant degradation of the structural element.

Both carbonation and the presence of chlorides are diffusion processes through the micropores of the concrete and are modeled as x = K √t, where x = penetration depth of the aggressive process, and K = coefficient that depends on the type of aggressive process, the characteristics of the material and the environmental conditions. Since all the elements analyzed do not have a significant presence of chlorides and are found in non-marine environments (corrosion not initiated by chlorides), the coefficient would be K = K${}_{c}$ due to carbonation alone and the time required for this to occur would be t${}_{i}$ = x${}^{2}$/K${}_{c}$${}^{2}$.

The carbonation coefficient is obtained as K${}_{c}$ = c${}_{env}$. c${}_{air}$. a. f${}_{cm}$${}^{b}$, where f${}_{cm}$ = average compressive strength of concrete in N/mm${}^{2}$, and is obtained from the tests carried out on the characteristic resistance f${}_{ck}$; f${}_{cm}$ = f${}_{ck}$ + 8,

c${}_{env}$ = environment coefficient, which is considered to be 1 if the element is protected from rain and 0.5 if it is exposed to rain,

c${}_{air}$ = air coefficient, which is considered to be 1 if the entrapped air is <4.5% and 0.7 if >4.5%. In all the cases studied, it is 1 since the elements studied are concretes executed in situ, except for building 8A, which is a precast concrete structure, and,

a,b = parameters depending on the type of binder (Portland Cement CEM1 and CEM2). In all cases a = 1800 and b = −1.7, according to table A.9.3 of the aforementioned Annex 9 of EHE [27] (Table 2).

The data obtained from the experimental phase in the laboratory and from the in situ tests, together with the coefficients in the application of the standard, offer the corresponding model of t${}_{i}$ = x${}^{2}$/K${}_{c}$${}^{2}$ for each element (Table 3) [27]. In this way, it is possible to know the period of initiation of corrosion of each element or pillar analyzed at a certain point in its useful life.

### 3.5. Corrosion Rate

Once the carbonation front has reached the reinforcement, the steel depassivates and corrosion can take place if oxygen and water are present. There is always a sufficient amount of oxygen that reaches the surface of the steel to allow the corrosion process. In this case, the corrosion rate is governed by the resistivity of the concrete (namely, the corrosion is under ohmic control).

Moisture content is the main factor determining the resistivity of carbonated concrete. Second, the microstructure of the concrete and the factors that determine it (type of cement, w/c ratio, curing, etc.) are also important for the carbonation rate and initiation time. The corrosion rate can be considered negligible, except for high humidity, or when the duration and frequency of the periods of water condensation on the concrete surface can cause variations in the moisture content at the level of the reinforcement.

The situation is much more serious than that described above if there are chlorides present in the concrete [49], even in such small quantities that by themselves, they would not give rise to corrosion. The presence of a small amount of chlorides in concrete can be due to the use of raw materials (water, aggregates) that contain these ions or to the penetration of chlorides from the external environment (seawater, de-icing salts).

In the cases studied, the existence of chlorides is negligible, and the corrosion rates contained in the standard model were considered [50].

### 3.6. Propagation of Corrosion Period

T${}_{p}$ = Propagation of corrosion period. The propagation stage ends when the loss in the reinforcement section is unacceptable or when cracks appear in the concrete covering. This situation occurred in all the cases of the elements studied.

The formula that includes the propagation time model according to point 1.2.3. of Annex 9 of EHE [27] models the propagation time as t${}_{p}$ = 80.x /∅.v${}_{corr}$, where:

- t${}_{p}$ = propagation time in years
- x = depth of concrete considered (reinforcement cover) in mm
- ∅ = reinforcement diameter in mm
- V${}_{corr}$ = rate of corrossion in μm/year

Corrosion rates were not measured, but in the absence of experimental data, 2 μm/year (0.17 mA/m${}^{2}$) was considered for IIb environments, and 3 μm/year (0.26 mA/m${}^{2}$) for IIa environments, in accordance with Table A.9.5 of the aforementioned Annex [27]. For the non-aggressive environments, a figure similar to that of IIb environments was considered for the effects of the corrosion rate in order to simulate a more unfavorable scenario. In all cases, moderate corrosion rates were considered according to RILEM (Table 4).

Likewise, all the t${}_{p}$ functions were calculated for each of the cases by modeling the propagation period according to the depth considered in the covering (x) and the corresponding diameter of each reinforcement (∅), which differed depending on the conditions of each building and the trusses of its pillars (Table 5).

### 3.7. Estimation of the Structure’s Useful Life in View of the Corrosion of the Reinforcements

Once the functions (initiation and propagation) had been calculated according to the EHE model in each of the cases, the sum of the total time of the two periods in the case of corrosion by carbonation would be: T = t${}_{i}$ + t${}_{p}$ = x${}^{2}$/K${}_{c}$${}^{2}$+ 80.x/∅.v${}_{corr}$, which is the total time considered as the period of the useful life of the building given the corrosion of the reinforcements.

**Figure 5:**
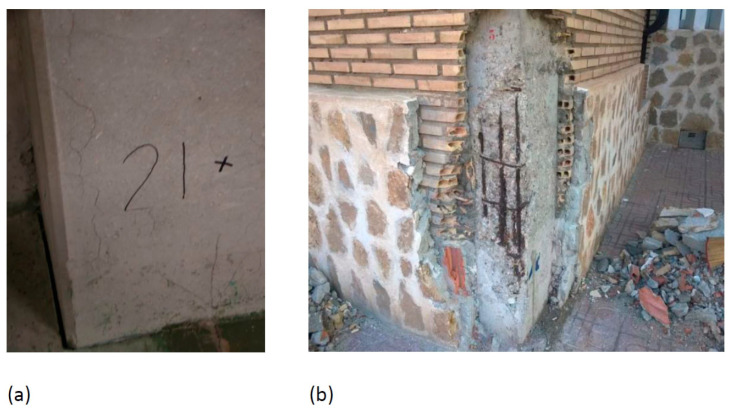
Corrosion pathologies. (**a**) Corrosion propagation period in Building 8 Pillar 21. The cracks parallel to the main reinforcements can be seen (**b**). Generalized corrosion. End of corrosion propagation and service life. Building 9 Pilar 1.

This working method incorporates all the data of each of the elements analyzed in the buildings in the sample and the models of the initiation times t${}_{i}$ (carbonation) and the propagation of corrosion t${}_{p}$, in such a way that the model of the expected useful life T = t${}_{i\;}$+ t${}_{p}$, together with the real data (age of the building), can be compared with the theoretical model considered by the standard (Figure 5).

## 4. Results

From the models (functions), we know the theoretical carbonation time (initiation of corrosion period or start time model t_*i*_) of the reinforcement coating and the expected useful life of each column analyzed from the position of its reinforcements (initiation time + corrosion propagation time), as well as the useful life model results (T = t_*i*_ + t_*p*_). Table 6 shows the results of all the cases studied in the buildings, which were between 9 and 44 years old.

In this chapter, the carbonation depths are also calculated from the data of each element and its behavioral model in order to make a comparison with the real carbonation depth. All the calculations were made from the graphical representation of the functions of the initiation models (initiation time t) and the total useful life (initiation plus propagation) using the GeoGebra Classic 5 program.

The results are the intersection points of the function with the position of the reinforcing bars (covering).

**Figure 6:**
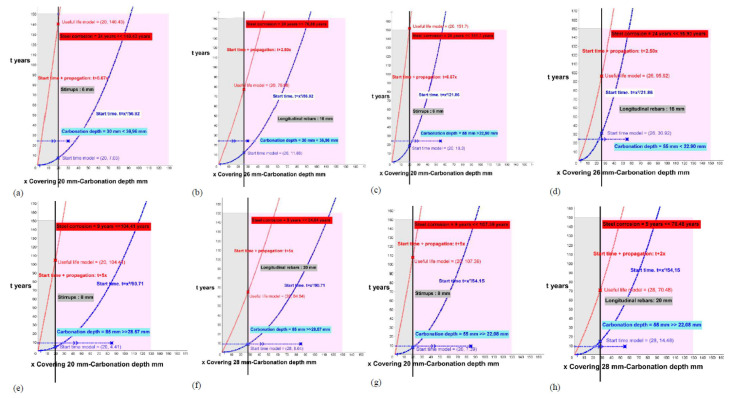
Start and propagation corrosion times in buildings 1D, 2G, 3T, 4T and 5T. (**a**) 1D_P8_Stirrups. (**b**) 1D_P8_ longitudinal rebars. (**c**) 1D_P16_Stirrups. (**d**) 1D_P16_ longitudinal rebars. (**e**) 2G_P1_ Stirrups. (**f**) 2G_P1_ longitudinal rebars. (**g**) 2G_P2_S Stirrups. (**h**) 2G_P2_ longitudinal rebars.

**Figure 7:**
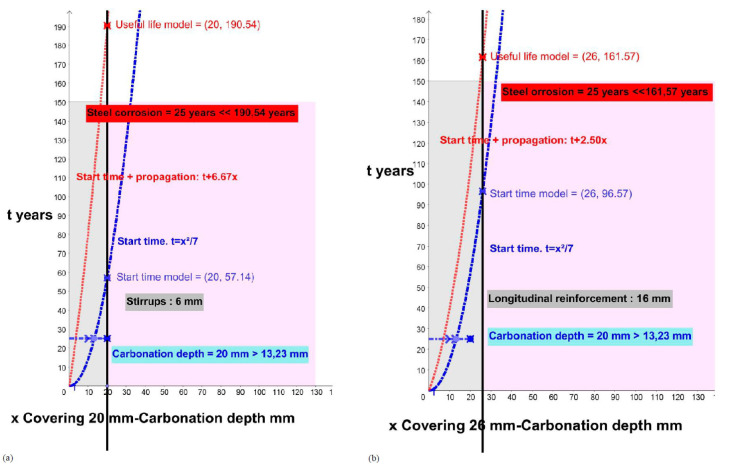
Start and propagation corrosion times in buildings 3T. **(a)** 3T_P2_ Stirrups. **(b)** 3T_P2_ longitudinal rebars.

**Figure 8:**
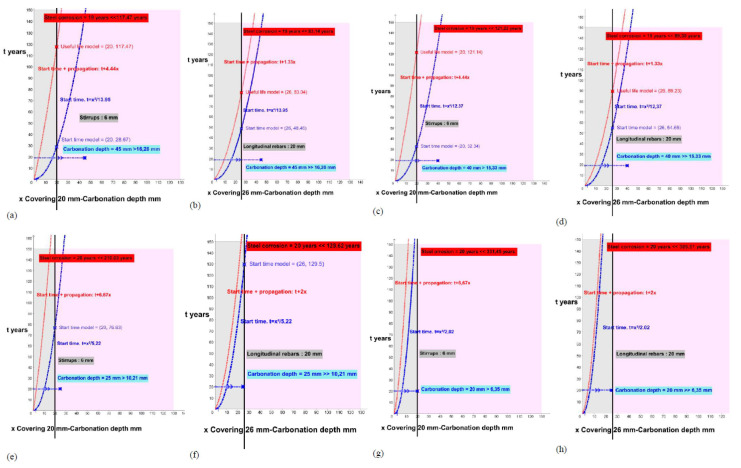
Start and propagation corrosion times in buildings 4T and 5T. (**a**) 4T_P2_ Stirrups. (**b**) 4T_P2_ longitudinal rebars. (**c**) 4T_P8_S Stirrups. (**d**) 4T_P8_ longitudinal rebars. (**e**) 5T_P3_ Stirrups. (**f**) 5T_P3_ longitudinal rebars. (**g**) 5T_P14_ Stirrups. (**h**) 5T_P14_ longitudinal rebars.

**Figure 9:**
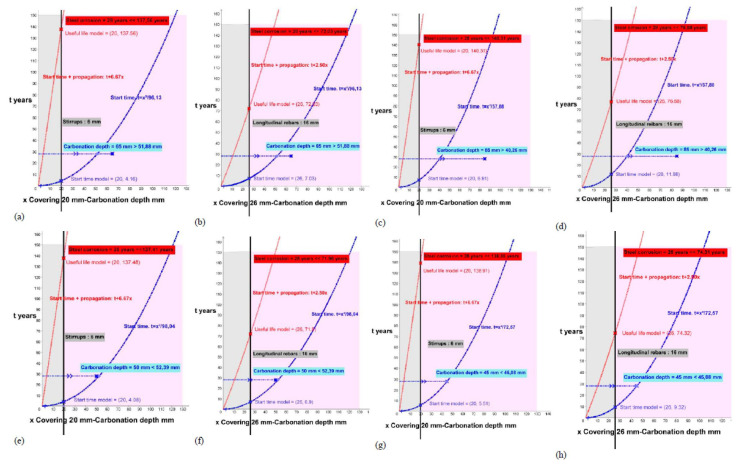
Start and propagation corrosion times in building 6D and 7R. (**a**) 6D_P1_ Stirrups. (**b**) 6D_P1_ longitudinal rebars. (**c**) 6D_P5_ Stirrups. (**d**) 6D_P5_ longitudinal rebars. (**e**) 7R_P1_ Stirrups. (**f**) 7R_P1_longitudinal rebars. (**g**) 7R_P2_ Stirrups. (**h**) 7R_P2_longitudinal rebars.

**Figure 10:**
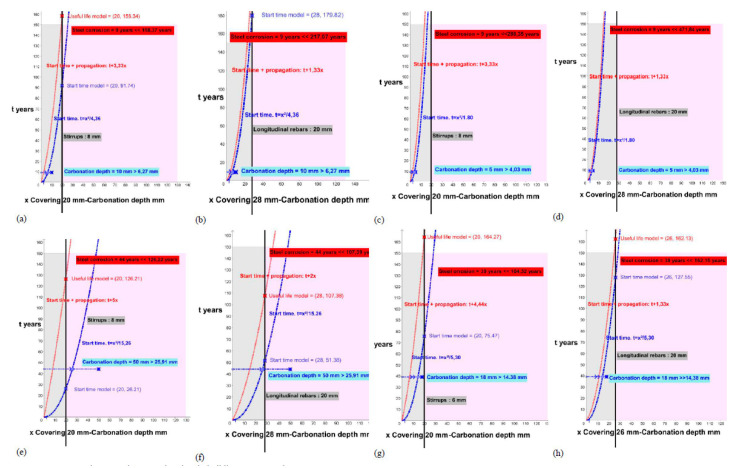
Start and propagation corrosion times in building 8A, 9M and 10E. (**a**) 8A_P1_ Stirrups. (**b**) 8A_P1_ longitudinal rebars. (**c**) 8A_P21_ Stirrups. (**d**) 8A_P21_longitudinal rebars. (**e**) 9M_P1_ Stirrups. (**f**) 9M_P1_ longitudinal rebars. (**g**) 10E_P11_Stirrups. (**h**) 10E_P11_longitudinal rebars.

This study processes each of the elements, for the initiation states (function of the blue curve) and the total time for the attack by carbonation for corrosion (function of the red curve), and offers us the results of the period (years) necessary to reach the carbonation of the entire coating (x in blue on the reinforcement) or the expected useful life of each analyzed element (x in red on the reinforcement) according to the theoretical models for the stirrups (Figure 6, Figure 7, Figure 8, Figure 9 and Figure 10; stapes in a, c, e, g) and transversal reinforcements (Figure 6, Figure 7, Figure 8, Figure 9 and Figure 10); longitudinal reinforcement in b, d, f, h). The results obtained are the solutions to the functions with the graphical representation in each of the cases (Figure 6, Figure 7, Figure 8, Figure 9 and Figure 10) and are also shown in the corresponding column in Table 6 (start time and useful life model).

The study also recorded the depth of carbonation in the concrete corresponding to the age at which the test was carried out. Once the mathematical expression of the corrosion initiation model is known, and taking advantage of the graphical analysis of each pillar studied, the carbonation depth corresponding to its age can be calculated, and thus it is possible to compare the result of the theoretical model (intersection of the horizontal blue line, which corresponds to the age of the element, with the blue function-curve corresponding to the initiation of corrosion) with the real depth obtained in the phenolphthalein test (x of the depth indicated as such on the horizontal blue line) of the 17 pillars in the 10 different buildings (Table 7).

Table 7 compares the actual carbonation suffered by the pillars with that which can be derived from the model established by the current Spanish regulations. Of these pillars, 11 present a carbonation that is between 192 and 315% higher than that derived from the model; in 3 of them, it is between 125 and 151% higher; while in the last 3 pillars, corresponding to low characteristic concrete strengths, it largely conforms to the regulatory model.

## 5. Discussion

**Figure 11:**
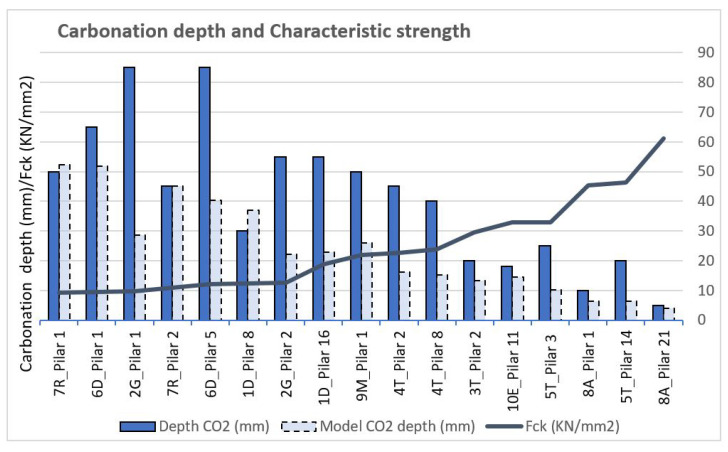
Carbonation depth and characteristic strength. Laboratory data and model data.

The results obtained from the depths of carbonation are provided in order to carry out a comparative study of the models and reality. The characteristic resistances of the 17 studied pillars are shown and the results are presented in order, from the lowest f${}_{ck}$ of the pillars to the highest, ranging from 9.30 KN/mm${}^{2}$ to a maximum of 61.10 KN/mm${}^{2}$, which is the most resistant concrete and corresponds to a structure of precast elements. The real carbonation depths were obtained in the range of 5 to 85 mm, while the predictions of the standard, considering the ages of each element, vary from 4.03 to 52.39 mm (Figure 11).

These results show a correspondence between the carbonation depth and the concrete strengths obtained, confirming that the carbonation depth decreases with increasing f${}_{ck}$, regardless of the age of the items. It reinforces the idea that the properties that influence carbonation related to the composition of the concrete (water/cement ratio, porosity and binder) are directly related to the strength of the concrete.

The depths of carbonation obtained in reality are, in most cases, greater than the depths of carbonation resulting from the application of the standard model.

**Figure 12:**
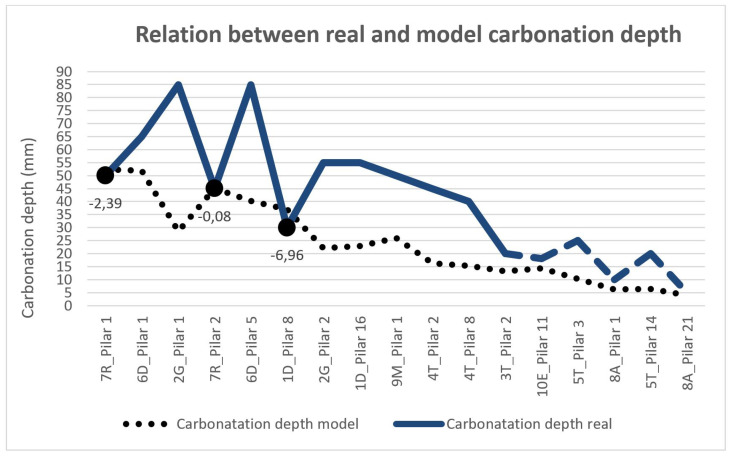
Real measurement (mm) and model depth carbonation (mm).

Figure 12 is a comparison of the real results and the figures derived from the model, and we can observe that in three cases, namely 7R_Pillar 1, 7R_Pillar 2 and 1D_Pillar 8, the real carbonation is less than or equal to the model’s figures, with values of 2.39, 0.08 and 6.96 mm, respectively, which is less than expected (points indicated in Figure 9). These are pillars with low f${}_{ck}$ and ages of approximately 24 years. In all the other cases, the carbonation depth of the concrete is greater than the estimated value.

Another result to consider and discuss occurred in the pillars with the highest resistance, of more than 30 KN/mm${}^{2}$ (3T_Pillar 2, 10E_Pillar 11, 5T_Pilar 3, 8A_Pillar 1, 5T_Pillar 14 and 8A_Pillar 21), in which the carbonation depth did not reach the reinforcement, which is between 20 and 28 mm, according to the transverse or longitudinal reinforcement coatings, respectively, and which corresponds to the graphical representation in the form of the dashed line in Figure 12. However, corrosion occurs [28,51]. This aspect will be the subject of continued investigation since the oxidation of steel is taking place in an alkaline environment in the pores of the concrete [52,53]. The results have also been expressed in increasing order of the characteristic strengths of the concrete. Therefore, this allows us to affirm that quality control in the production site of concrete results in a lower carbonation depth [54,55]. This is very evident in building 8A, which was built entirely with a precast reinforced concrete structure, resulting in the highest characteristic strength and the lowest carbonation depth of all of the researched buildings.

For the discussion of the data obtained in the corrosion initiation and propagation models, the useful life of the element is considered, analyzing the data in longitudinal reinforcements, where carbonation (initiation) arrives later due to a thicker coating than in the stirrups (transversal reinforcement), but the propagation of corrosion progresses more quickly since it depends on the reinforcement’s diameter and it has a larger section ∅. Furthermore, in most cases, the most obvious pathologies originate from the oxidation of the main reinforcements, producing cracks up to the concrete surface parallel to its trajectory. That is why the results in Table 6, referring to the main reinforcements, are shown.

**Figure 13:**
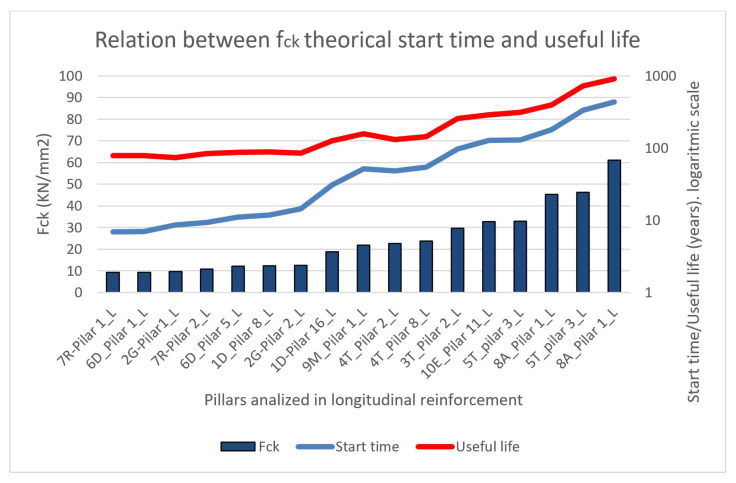
The relative study of F${}_{ck}$, theoretical start time and theoretical useful life.

The data are presented in order from lowest to highest characteristic strength (Figure 13). The results of the theoretical models of the corrosion initiation periods express a clear tendency toward longer carbonation times of the coating for higher f${}_{ck}$, which is in accordance with the mathematical expressions contemplated by the regulations. The same happens with the periods of propagation of corrosion and, therefore, with the theoretical useful life (sum of beginning and propagation), which increases considerably with the characteristic resistance of the concrete of the element.

As the strength of the concrete increases (Figure 13), the difference between the service life model and the starting period, that is, the period of corrosion propagation, decreases slightly. In element 7R-Pillar1, the model of the corrosion propagation period is 65 years (71.90–6.90), and in 8A-pillar 21 it is 37 years (471.84–434.51). The propagation periods decrease with the increase in the strength of the concrete. However, according to the existing pathologies and current ages, the propagation periods were much shorter.

**Figure 14:**
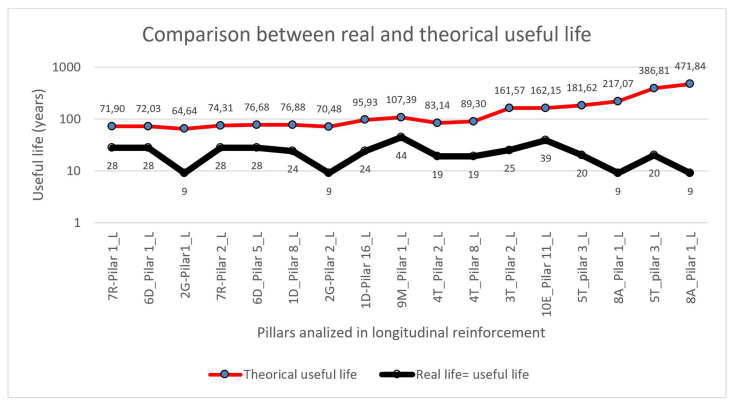
Relation between real and theoretical useful life.

In Figure 14, following the same order of presentation for the results (according to f${}_{ck}$ from lowest to highest), the results of the useful life model and the real life of the element are shown, and it is clear that the real useful life (between 9 and 44 years) is much lower than that expected (from 71 to 471 years).

## 6. Conclusions

The processes of corrosion of the reinforcements in reinforced concrete are very complex, and their analysis can be undertaken from the application of the mathematical models offered by the regulations. They have been studied in 10 residential buildings in non-aggressive and normal environments, and in the verified absence of chlorides, with the structural elements only being exposed to corrosion by carbonation. All the cases studied are centered on reinforced concrete pillars on the lower floors of the building and in which corrosion of the reinforcements had occurred with evident pathology (cracks on their surface, parallel to the direction of the longitudinal reinforcements). The structures cannot be considered old since none of the buildings was more than 50 years old, which is less than the minimum useful life that a residential building should have.

The studies of the depth of carbonation reflect a behavior that is clearly related in a linear way: as the strength of the concrete increases, the depth of carbonation decreases. However, this carbonation is much higher than that estimated by the standard models for the environments studied, in some cases by around 300%. Only in three of the seventeen cases was the extent of carbonation similar to that expected, and, in six of the most resistant concretes, the depth of carbonation was less than the thickness of the coatings of the reinforcements. Despite this, all the elements studied had been attacked by corrosion of the reinforcements. This indicates the difficulty in understanding the process and alerts us to the existence of other oxidation mechanisms that can cause a galvanic cell in the Fe or reinforcement that releases ferrous and ferric ions, or even to other types of oxidation generated by other electrolytic processes, which opens up a new field of research. Two possible causes are considered:

(a) In the cases of higher levels of carbonation, different levels of humidity were detected between the lower part (humidity by capillarity or from an external source) and the upper part of the element. The standard models do not consider this fact, and this suggests an area that could be explored.

(b) In the cases of lower levels of carbonation (corresponding to higher characteristic strength), there were no appreciable differences in humidity, so the causes can be attributed to a different electrochemical potential between products at the top and bottom of the pillar.

Regardless of the differences in the real age, there is a clear and evident shortening of the useful life of the element, compared with the model that results from the application of the standard (the initiation and propagation periods of corrosion decrease). This situation is the consequence of an unexpected increase in the depth of carbonation in most cases, and in other cases, it is caused by processes that are independent of the alkalinity of the medium and which are presumably produced by other electrochemical processes. We can conclude that the Spanish standard EHE 08 needs a thorough revision, which should include other variables related to corrosion processes in the models for obtaining corrosion initiation and propagation periods. Among other factors, it is necessary to consider the humidity caused by capillarity and the differences in electrochemical potential between materials and different construction systems.

## Figures and Tables

**Table 1:** General exposure classes related to rebar corrosion.

General Exposure Class				Description	Examples
Class	Subclass	Designation	Process Type		
**Non-aggressive**		**I**	Nothing	Interiors of buildings, not subjected to condensation. Mass concrete elements.	Structural elements of buildings, including floors that are protected from the elements.
**Normal**	High humidity	**IIa**	Corrosion not caused by chlorides	Interiors subject to high average relative humidity (>65%) or condensation. Exteriors in the absence of chlorides and exposed to rain in areas with average annual rainfall greater than 600 mm. Buried or submerged elements.	Structural elements in non-ventilated basements. Foundations, bridge abutments, piers and decks in unsealed areas with average annual rainfall greater than 600 mm. Waterproofed bridge decks, in areas with de-icing salts or average annual rainfall greater than 600 mm. Concrete elements found outdoors or on the roofs of buildings in areas with average annual rainfall greater than 600 mm. Forged elements in a sanitary chamber or in the interior of kitchens and bathrooms or on unprotected roofs.
	Medium humidity	**IIb**	Corrosion not caused by chlorides	Exteriors in the absence of chlorides subjected to the action of rainwater in areas with average annual rainfall less than 600 mm.	Structural elements in outdoor buildings protected from rain. Bridge decks and piers in areas with average annual rainfall less than 600 mm.

**Table 2:** Parameters depending on the type of binder.

Binder	Instruction Cement RC 03	a	b
Portland Cement	CEM I CEM II/A CEM/II/B-S CEM II/B-L CEM II/B-LL CEM II/B-M CEM/V	1800	−1.7
Portland Cement + 28% fly ash	CEM II/B-P CEM II/B-V CEM IV/A CEM IV/B	360	−1.2
Portland Cement + 9% silica fume	CEM II/A-D	400	−1.2
Portland Cement + 65% slag	CEM III/A CEM III/B	360	−1.2

**Table 3:** Model of the corrosion initiation period of each element.

Environment	Building	C${}_{\mathrm{env}}$	C${}_{\mathrm{air}}$	a	b	f${}_{\mathrm{ck}}$	f${}_{\mathrm{cm}}$ = f${}_{\mathrm{ck}}$ + 8	K${}_{c}$ = c${}_{\mathrm{env}}$. c${}_{\mathrm{air}}$. a. f${}_{\mathrm{cm}}$${}^{b}$	ti = x^2^/Kc^2^
**I**	**1D**								
I	1D_Pillar 8_interior_Level 0	1.00	0.70	1800	−1.70	12.30	20.30	7.54	ti = x${}^{2}$/56.92
I	1D_Pillar 16_int. wall_Level 0	1.00	0.70	1800	−1.70	18.90	26.90	4.68	ti = x${}^{2}$/21.86
**I**	**2G**								
I	2G_Pillar 1_interior_Level 0	1.00	0.70	1800	−1.70	9.70	17.70	9.52	ti = x${}^{2}$/90.71
I	2G_Pillar 2_exterior wall_Level 0	1.00	0.70	1800	−1.70	12.60	20.60	7.36	ti = x${}^{2}$/54.15
**I**	**3T**								
I	3T_Pillar 2_interior wall_Level 0	1.00	0.70	1800	−1.70	29.60	37.60	2.65	ti = x${}^{2}$/7
**IIa**	**4T**								
IIa	4T_Pillar 2_interior_basement	1.00	0.70	1800	−1.70	22.70	30.70	3.73	ti = x${}^{2}$/13.95
IIa	4T_Pillar 8_int._basement	1.00	0.70	1800	−1.70	23.80	31.80	3.52	ti = x${}^{2}$/12.37
**I**	**5T**								
I	5T_Pillar 3_exterior wall_level 0	1.00	0.70	1800	−1.70	33.00	41.00	2.28	ti = x${}^{2}$/5.22
I	5T_Pillar 14_exterior wall_level 0	1.00	0.70	1800	−1.70	46.2	54.20	1.42	ti = x${}^{2}$/2.02
**I, II**	**6D**								
IIb	6D_Pillar 1_exterior wall_level 0	1.00	0.70	1800	−1.70	9.40	17.40	9.80	ti = x${}^{2}$/96.13
I	6D_Pillar 5_interior_level 0	1.00	0.70	1800	−1.70	12.20	20.20	7.61	ti = x${}^{2}$/57.88
**I**	**7R**								
I	7R_Pillar 1_Interior_level 0	1.00	0.70	1800	−1.70	9.30	17.30	9.90	ti = x${}^{2}$/98.04
I	7R_Pillar 2_Interior_level 0	1.00	0.70	1800	−1.70	10.90	18.90	8.52	ti = x${}^{2}$/72.57
**II**	**8A**								
IIa	8A_Pillar 1_interior_basement	1.00	1.00	1800	−1.70	45.30	53.30	2.09	ti = x${}^{2}$/4.36
IIa	8A_Pillar 21_interior_basement	1.00	1.00	1800	−1.70	61.10	69.10	1.34	ti = x${}^{2}$/1.80
**I**	**9M**								
I	9M_Pillar 1_exterior wall_level 0	1.00	0.70	1800	−1.70	21.90	29.90	3.91	ti = x${}^{2}$/15.26
**II**	**10E**								
IIa	10E_Pillar 11_interior_basement	1.00	0.70	1800	−1.70	32.80	40.80	2.30	ti = x${}^{2}$/5.30

**Table 4:** Corrosion rate according to general exposure classes.

General Exposure Class			V_*corr*_ (μm/year)
Normal	High humidity	IIa	3
	Medium humidity	IIb	2

**Table 5:** Model of the corrosion propagation period in each reinforcement.

Ambient	Building	∅	Vcorr	t${}_{p}$ = 80.x /∅.v_*corr*_
**I**	**1D**			
I	Stirrups	6	2	tp = 6.67x
I	Longitudinal rebars	16	2	tp = 2.5x
**I**	**2G**			
I	Stirrups	8	2	tp = 5x
I	Longitudinal rebars	20	2	tp = 2x
**I**	**3T**			
I	Stirrups	6	2	tp = 6.67x
I	Longitudinal rebars	16	2	tp = 2.5x
**IIa**	**4T**			
IIa	Stirrups	6	3	tp = 4.44x
IIa	Longitudinal rebars	20	3	tp = 1.33x
**I**	**5T**			
I	Stirrups	6	2	tp = 6.67x
I	Longitudinal rebars	20	2	tp = 2x
**I, II**	**6D**			
IIb	Stirrups	6	2	tp = 6.67x
IIb	Longitudinal rebars	16	2	tp = 2.5x
**I**	**7R**			
I	Stirrups	6	2	tp = 6.67x
I	Longitudinal rebars	16	2	tp = 2.5x
**II**	**8A**			
IIa	Stirrups	8	3	tp = 3.33x
IIa	Longitudinal rebars	20	3	tp = 1.33x
**I**	**9M**			
I	Stirrups	8	2	tp = 5x
I	Longitudinal rebars	20	2	tp = 2x
**II**	**10E**			
IIa	Stirrups	6	3	tp = 4.44x
IIa	Longitudinal rebars	20	3	tp = 1.33x

**Table 6:** Studied buildings. Initiation (start time) and propagation time corrosion model. Useful life model.

Environment	Building	f${}_{ck}$	K ${}_{c}$	Start Time t ${}_{i}$	Start Time Model (Covering)	∅ mm	Propagation Time t ${}_{p}$	Useful Life Model (T = t${}_{i}$ + t${}_{p}$) (Covering)	Age (Year)
**I**	**1D-Calle General Pastor, 19, Dolores.**								
I	1D_Pillar 8_interior_Level 0/Stirrups	12.30	7.54	**t** ${}_{i}$ ** = x${}^{2}$/56.92**	7.03	6	**t** ${}_{p}$ ** = 6.67x**	140.36	24
I	1D_Pillar 8_interior_Level 0/Longitudinal rebars	12.30	7.54	**t** ${}_{i}$ ** = x${}^{2}$/56.92**	11.88	16	**t** ${}_{p}$ ** = 2.5x**	76.88	24
I	1D_Pillar 16_int. wall_Level 0/Stirrups	18.90	4.68	**t** ${}_{i}$ ** = x${}^{2}$/21.86**	18.30	6	**t** ${}_{p}$ ** = 6.67x**	151.63	24
I	1D-Pillar 16_int.wall_Level 0/Longitudinal rebars.	18.90	4.68	**t** ${}_{i}$ ** = x${}^{2}$/21.86**	30.93	16	**t** ${}_{p}$ ** = 2.5x**	95.93	24
**I**	**2G-Calle San Pedro, 38, Guardamar**								
I	2G_Pillar 1_interior_Level 0/Stirrups	9.70	9.52	**t** ${}_{i}$ ** = x${}^{2}$/90.71**	4.41	8	**t** ${}_{p}$ ** = 5x**	104.41	9
I	2G_Pillar 1_interior_Level 0/ Longitudinal rebars	9.70	9.52	**t** ${}_{i}$ ** = x${}^{2}$/90.72**	8.64	20	**t** ${}_{p}$ ** = 2x**	64.64	9
I	2G_Pillar 2_ext. wall_Level 0/Stirrups	12.60	7.36	**t** ${}_{i}$ ** = x${}^{2}$/54.15**	7.39	8	**t** ${}_{p}$ ** = 5x**	107.39	9
I	2G_Pillar 2_ext. wall_Level 0/ Longitudinal rebars	12.60	7.36	**t** ${}_{i}$ ** = x${}^{2}$/54.15**	14.48	20	**t** ${}_{p}$ ** = 2x**	70.48	9
**I**	**3T-Calle Ulpiano,71, Torrevieja**								
I	3T_Pillar 2_interior wall_Level 0/Stirrups	29.60	2.65	**t** ${}_{i}$ ** = x${}^{2}$/7**	57.14	6	**t** ${}_{p}$ ** = 6.67x**	190.48	25
I	3T_Pillar 2_int. wall_Level 0/Longitudinal rebars	29.60	2.65	**t** ${}_{i}$ ** = x${}^{2}$/7**	96.57	16	**t** ${}_{p}$ ** = 2.5x**	161.57	25
**IIa**	**4T-Av. Holandeses, Torrevieja.**								
IIa	4T_Pillar 2_interior_basement/Stapes	22.70	3.73	**t** ${}_{i}$ ** = x${}^{2}$/13.95**	28.68	6	**t** ${}_{p}$ ** = 4.44x**	117.57	19
IIa	4T_Pillar 2_int._basement/Longitudinal rebars	22.70	3.73	**t** ${}_{i}$ ** = x${}^{2}$/13.95**	48.47	20	**t** ${}_{p}$ ** = 1.33x**	83.14	19
IIa	4T_Pillar 8_int._basement/Stirrups	23.80	3.52	**t** ${}_{i}$ ** = x${}^{2}$/12.37**	32.33	6	**t** ${}_{p}$ ** = 4.44x**	121.22	19
IIa	4T_Pillar 8_int._basement/Longitudinal rebars	23.80	3.52	**t** ${}_{i}$ ** = x${}^{2}$/12.37**	54.63	20	**t** ${}_{p}$ ** = 1.33x**	89.30	19
**I**	**5T- Av. Inglaterra, 31, Torrevieja**								
I	5T_Pillar 3 _ext. wall_level 0/Stirrups	33.00	2.28	**t** ${}_{i}$ ** = x${}^{2}$/5.22**	76.70	6	**t** ${}_{p}$ ** = 6.67x**	210.03	20
I	5T_Pillar 3_ext wall_Level 0/Longitudinal rebars	33.00	2.28	**t** ${}_{i}$ ** = x${}^{2}$/5.22**	129.62	20	**t** ${}_{p}$ ** = 2x**	181.62	20
I	5T_Pillar 14_ext wall_level 0/Stirrups	46.20	1.42	**t** ${}_{i}$ ** = x${}^{2}$/2.02**	198.11	6	**t** ${}_{p}$ ** = 6.67x**	331.45	20
I	5T_Pillar 14_ext wall_Level 0/Longitudinal rebars	46.20	1.42	**t** ${}_{i}$ ** = x${}^{2}$/2.02**	334.81	20	**t** ${}_{p}$ ** = 2x**	386.81	20
**I, II**	**6D-Av. Crevillente, 4, Dolores**								
IIb	6D_Pillar 1_ext. wall_level 0/ Stirrups	9.40	9.80	**t** ${}_{i}$ ** = x${}^{2}$/96.13**	4.16	6	**t** ${}_{p}$ ** = 6.67x**	137.49	28
IIb	6D_Pillar 1_ext. wall_level 0/ Longitudinal rebars	9.40	9.80	**t** ${}_{i}$ ** = x${}^{2}$/96.13**	7.03	16	**t** ${}_{p}$ ** = 2.5x**	72.03	28
I	6D_Pillar 5_interior_level 0/ Stirrups	12.20	7.61	**t** ${}_{i}$ ** = x${}^{2}$/57.88**	6.91	6	**t** ${}_{p}$ ** = 6.67x**	140.24	28
I	6D_Pillar 5_interior_level 0/Longitudinal rebars	12.20	7.61	**t** ${}_{i}$ ** = x2/57.88**	11.68	16	**t** ${}_{p}$ ** = 2.5x**	76.68	28
**I**	**7R-Pl. Héroes de Africa, Rojales.**								
I	7R_Pillar 1_Interior_level 0/ Stirrups	9.30	9.90	**t** ${}_{i}$ ** = x${}^{2}$/98.04**	4.08	6	**t** ${}_{p}$ ** = 6.67x**	137.41	28
I	7R_Pillar 1_interior_level 0/Longitudinal rebars	9.30	9.90	**t** ${}_{i}$ ** = x${}^{2}$/98.04**	6.90	16	**t** ${}_{p}$ ** = 2.5x**	71.90	28
I	7R_Pillar 2_Interior_level 0/ Stirrups	10.90	8.52	**t** ${}_{i}$ ** = x${}^{2}$/72.57**	5.51	6	**t** ${}_{p}$ ** = 6.67x**	138.85	28
I	7R_Pillar 2int._level 0/Longitudinal rebars	10.90	8.52	**t** ${}_{i}$ ** = x${}^{2}$/72.57**	9.31	16	**t** ${}_{p}$ ** = 2.5x**	74.31	28
**II**	**8A-Av. La Peseta, 19, Alicante.**								
IIa	8A_Pillar 1_interior_basement/ Stirrups	45.30	2.09	**t** ${}_{i}$ ** = x${}^{2}$/4.36**	91.70	8	**t** ${}_{p}$ ** = 3.33x**	158.37	9
IIa	8A_Pillar_int._basement/Longitudinal rebars	45.30	2.09	**t** ${}_{i}$ ** = x${}^{2}$/4.37**	179.74	20	**t** ${}_{p}$ ** = 1.33x**	217.07	9
IIa	8A_Pillar 21_int._basement/ Stirrups	61.10	1.34	**t** ${}_{i}$ ** = x${}^{2}$/1.80**	221.69	8	**t** ${}_{p}$ ** = 3.33x**	288.35	9
IIa	8A_Pillar 21_int._basement/Longitudinal rebars	61.10	1.34	**t** ${}_{i}$ ** = x${}^{2}$/1.80**	434.51	20	**t** ${}_{p}$ ** = 1.33x**	471.84	9
**I**	**9M-Calle Iberia, 33, Aguilas. Murcia**								
I	9M_Pillar 1_ext. wall_level 0/Stirrups	21.90	3.91	**t** ${}_{i}$ ** = x${}^{2}$/15.26**	26.22	8	**t** ${}_{p}$ ** = 5x**	126.22	44
I	9M_Pillar 1_ext. wall_level 0/Longitudinal rebars	21.90	3.91	**t** ${}_{i}$ ** = x${}^{2}$/15.26**	51.39	20	**t** ${}_{p}$ ** = 2x**	107.39	44
**II**	**10E-Av. San Bartolomé de T. 8, Elche.**								
IIa	10E_Pillar 11_int._basement/ Stirrups	32.80	2.30	**t** ${}_{i}$ ** = x${}^{2}$/5.30**	75.43	6	**t** ${}_{p}$ ** = 4.44x**	164.32	39
IIa	10E_Pillar 11_int._basement/Longitudinal rebars	32.80	2.30	**t** ${}_{i}$ ** = x${}^{2}$/5.30**	127.48	20	**t** ${}_{p}$ ** = 1.33x**	162.15	39

**Table 7:** Real and model carbonation depths corresponding to age in each element.

Environment	Building-Element	f${}_{ck}$ (KN/mm${}^{2}$)	Carbonation Depth (mm)	Carbonation Depth Model (mm)	Difference (mm)	Age (years)	Increased Carbonation (%)
I	7R_Pillar 1	9.30	50	52.39	−2.39	28	95.4
IIb	6D_Pillar 1	9.40	65	51.88	13.12	28	125.3
I	2G_Pillar 1	9.70	85	28.57	56.43	9	297.5
I	7R_Pillar 2	10.90	45	45.08	−0.08	28	100
I	6D_Pillar 5	12.20	85	40.26	44.74	28	211.1
I	1D_Pillar 8	12.30	30	36.96	−6.96	24	81.2
I	2G_Pillar 2	12.60	55	22.08	32.92	9	249.1
I	1D_Pillar 16	18.90	55	22.90	32.10	24	240.2
I	9M_Pillar 1	21.90	50	25.91	24.09	44	192.9
IIa	4T_Pillar 2	22.70	45	16.28	28.72	19	276.4
IIa	4T_Pillar 8	23.80	40	15.33	24.67	19	260.9
I	3T_Pillar 2	29.60	20	13.23	6.77	25	151.2
IIa	10E_Pillar 11	32.80	18	14.38	3.62	39	125.2
I	5T_Pillar 3	33.00	25	10.21	14.79	20	244.9
IIa	8A_Pillar 1	45.30	10	6.27	3.73	9	159.5
I	5T_Pillar 14	46.2	20	6.35	13.65	20	315
IIa	8A_Pillar 21	61.10	5	4.03	0.97	9	124

## Data Availability

The data presented in this study are available on request from the corresponding author.
